# Role of triglycerides as a predictor of autoimmune hepatitis with cirrhosis

**DOI:** 10.1186/s12944-022-01716-9

**Published:** 2022-10-25

**Authors:** Peng Wang, Yuqi Wang, Hui Liu, Xiaoxu Han, Yunyun Yi, Xin Wang, Xin Li

**Affiliations:** 1 Center of Integrative Medicine, Beijing Ditan Hospital, Capital Medical University, No. 8 Jing Shun East Street, Beijing, 100015 China; 2 Center of Integrative Medicine, Peking University Ditan Teaching Hospital, Beijing, 100015 China

**Keywords:** Autoimmune hepatitis, cirrhosis, triglyceride, predictor

## Abstract

**Background:**

Metabolism-related indicators have been suggested as possible prognostic indicators of liver disease in recent relevant studies, but their value in predicting autoimmune hepatitis (AIH) cirrhosis is unclear. This study evaluated the role of lipid levels in determining the prognosis of AIH-related cirrhosis.

**Methods:**

We retrospectively included 345 patients with AIH who were initially diagnosed at Beijing Ditan Hospital from 2010-2019, and ultimately screened 196 patients who met the criteria. A logistic regression analysis was performed to screen factors associated with cirrhosis. Kaplan–Meier (KM) curves were constructed to analyze the effects of different triglyceride (TG) levels on the survival of patients with cirrhosis. A restricted cubic spline fitted Cox regression model was used to analyze the nonlinear relationship between serum TG levels and patient prognosis.

**Results:**

Patients with AIH cirrhosis have lower TG levels than those without cirrhosis. Lower serum TG levels correlated with the severity of cirrhosis. The survival analysis showed that TG levels were associated with the overall survival of patients with AIH, as a lower 5-year survival rate (log-rank *P*<0.05) was observed for patients in the TG≤0.95 mmol/L group (hazard ratio (HR)=3.79, 95% CI: 1.528-9.423). In addition, lower TG levels were associated with a higher incidence of death in patients with AIH cirrhosis. The risk of death gradually increased for the interval of TG levels of 0.5-0.8 mmol/L (*P* for nonlinearity<0.001), and the hazard ratio per standard deviation increase in the TG level was 0.97 (95% CI: 0.94-0.99). The plot showed a U-shaped relationship between TG levels and the survival of patients with decompensated cirrhosis. The risk ratio progressively decreased with lower TG levels (*P* for nonlinearity=0.002). Below 0.6 mmol/L, the probability of TG risk per standard deviation prediction was 1.49 (95% CI: 1.00-2.24).

**Conclusion:**

Serum TG levels are closely related to the disease severity and overall survival of patients with AIH cirrhosis and may be used as a new indicator of advanced liver disease and long-term prognosis.

## Introduction

Autoimmune hepatitis (AIH) is a rare chronic autoimmune disease that is closely related to genetic components and environmental factors [1]. The exact pathogenesis of AIH is not yet fully understood. Patients with AIH are prone to develop cirrhosis or end-stage liver disease if clinical intervention is not performed. Due to the insidious onset, approximately one-third of adult AIH patients have cirrhosis at the time of diagnosis [2]. Previous studies have confirmed that cirrhosis is strongly associated with a poor prognosis and progression to end stage liver diseases in patients with AIH [3–5]. Therefore, close monitoring of risk factors affecting the development and progression of cirrhosis as well as aggressive treatment are essential to reduce liver-related deaths and improve the quality of life of patients with AIH [6].

The liver plays a crucial role in lipid synthesis and metabolism in humans, including the synthesis and degradation of total cholesterol (TC), triglycerides (TGs) and lipoproteins. The development of liver-related diseases may seriously affect metabolic function [7]. Several studies have shown that dyslipidemia occurs in patients with a variety of liver diseases and may be considered a new predictor of liver prognosis. In individuals with nonalcoholic fatty liver disease (NAFLD), lipoproteins and TGs are important risk factors for the development of hepatocellular carcinoma (HCC) in patients with cirrhosis [8]. High-density lipoprotein cholesterol (HDL-C), a lipid index, influences the predictive factors for the development of and death related to hepatitis B virus (HBV) cirrhosis, hepatitis C virus (HCV) cirrhosis and noncholestatic cirrhosis [9, 10]. Other studies have shown that TG levels are reduced in patients with hepatitis B-related cirrhosis and HCC, and that lower TG levels affect the prognosis of patients [11, 12]. In addition, TC and low-density lipoprotein cholesterol (LDL-C) levels are significantly reduced in patients with advanced chronic liver disease including HBV, fatty liver and AIH [13, 14]. The changes in these metabolic parameters and their effects on patient prognosis may be attributed to the decrease in hepatic lipid synthesis capacity. However, hepatic metabolic levels are still rarely studied among patients with AIH, and the relationship between metabolic indicators and the development or prognosis of AIH or AIH related cirrhosis is unclear.

In this study, we observed changes in lipid levels in patients with AIH-related cirrhosis, and then analyzed the correlation of metabolism-related indicators with disease progression and outcomes in patients to assess their potential as prognostic indicators in patients with AIH related cirrhosis.

## Materials and methods

### Patients

This study retrospectively included 345 patients with initially diagnosed AIH who were admitted to Beijing Ditan Hospital affiliated with Capital Medical University, from 2010 to 2019. The diagnosis of AIH was based on criteria established by international panels. Patients were diagnosed with AIH by adhering to the revised 2008 simplified scoring criteria from the International Autoimmune Hepatitis Group [15, 16], and those with scores ≥6 were included in this study. Patients under the age of 18 years (*n*=5); combined with other liver diseases such as viral hepatitis (*n*=7), nonalcoholic fatty liver disease (*n*=16), drug-induced liver injury (*n*=61), and primary biliary cholangitis (*n*=54); incomplete data (*n*=6) ; and use of hypolipidemic agents within 6 months were excluded from this study. A total of 196 patients with AIH meeting the criteria were finally included. This study was approved by the Ethics Committee of Beijing Ditan Hospital.

### Data collection and definition

Patient data were collected mainly from medical records in the hospital system, including baseline demographic characteristics (age, sex, basic diseases), routine biochemical parameters (white blood cell (WBC), red blood cell (RBC), gamma-glutamyltranspeptidase (GGT), total bilirubin (TBIL), hemoglobin (HGB), platelets (PLTs), international normalized ratio (INR), prothrombin activity (PTA), alanine aminotransferase (ALT), and aspartate aminotransferase (AST) levels), autoimmune disease-related antibodies and indicators (immunoglobulin G (IgG), immunoglobulin A (IgA), immunoglobulin M (IgM), anti-nuclear antibodies (ANA), and anti-smooth muscle antibodies (SMA)), serum lipid parameters (TG, TC, HDL-C, and LDL-C), histology and imaging data. Child–Pugh scores and the model for end-stage liver disease (MELD) scores were calculated based on specific variables [17, 18]. Cirrhosis was diagnosed based on definitive imaging and histology. Other concomitant autoimmune diseases included Hashimoto's thyroiditis, systemic lupus erythematosus, Sjogren's syndrome, and rheumatoid arthritis. Hepatic histological indications for AIH were interface type hepatitis, lymphocytic or plasma cell infiltration, rosette-like formation, and penetration phenomenon [19–22]. Overall survival (OS) was defined as the time from randomization to death due to any cause.

### Statistical analysis

All data were statistically analyzed using SPSS version 22 and GraphPad version 8.0. Statistical significance was defined as a *P* value less than 0.05. For comparisons between two groups, the chi-square test was used for categorical variables (reported as percentages and values), the T test was used for continuous variables with a normal distribution (means ± standard deviations), and the Mann–Whitney U test (median ± quartile) was used for continuous variables with a nonnormal distribution. Univariate and multivariate regression analyses were performed using binary logistic models to assess factors associated with cirrhosis. The cutoff value was defined as the maximum Youden index. Survival curves and restricted cubic splines were plotted using R version 4.2. Survival curves were constructed to compare overall survival between the two groups using the log-rank test. Cubic spline was used to analyze the nonlinear relationship between the index and overall survival by fitting a Cox proportional risk model with 5 nodes or 3 nodes of TG levels, and a likelihood ratio test was used to test the potential nonlinearity.

## Results

### Baseline characteristics of cirrhosis and noncirrhosis AIH patients

A total of 196 patients with AIH were enrolled in the study, including 168 women and 28 men, all with an average age of 56 years. Forty-three patients with AIH were complicated with other autoimmune diseases, including 9 patients with Sjogren's syndrome, 14 patients with Hashimoto's thyroiditis, 10 patients with rheumatoid arthritis, 5 patients with systemic lupus erythematosus and 5 patients with atopic dermatitis. In addition, 80 patients with cirrhosis and 116 noncirrhosis patients were included in the total population. Table 1 shows the baseline characteristics of the two groups, including demographic, liver function, and lipid profiles. Patients with cirrhosis were older than those without cirrhosis. In addition, patients with cirrhosis also had higher levels of IgA, white blood cells, hemoglobin and INR. However, PTA, PLT and GGT levels were higher in the noncirrhotic group. The blood lipid levels of patients were also analyzed, including TG, TC, HDL-C and LDL-C, and patients with cirrhosis had lower levels of TG, TC and LDL-C (*P*<0.05). The two groups were similar in sex, other autoimmune diseases and diabetes mellitus.

**Table 1:** Baseline characteristics of patients with AIH cirrhosis and noncirrhotic patients

	Noncirrhosis (*n*=116)	Cirrhosis (*n*=80)	*P* values
Age (mean±SD)	51 ± 15	62 ± 12	<0.001
Female (%)	100 (86.2%)	68 (85%)	0.812
Diabetes (%)	17 (14%)	18 (23%)	0.159
Hypertension (%)	27 (23%)	15 (19%)	0.448
ANA or SMA (%)	111 (96%)	78 (98%)	0.245
ALT (U/L)	232.7 ± 198.9	100.8 ± 178.5	<0.001
AST (U/L)	212.1 ± 176.9	139.9 ± 222.2	0.013
ALP/AST	1.6 ± 2.2	4.0 ± 4.3	<0.001
TBIL (mg/dL)	46.8 (18.5,122.3)	36.1 (15.9,109.4)	0.321
GGT (U/L)	193.8 ± 180.1	121.2 ± 134.8	0.003
IgG (g/L)	23.4 (20.1,29.2)	25.3 (20.5,33.1)	0.169
IgA (g/L)	3.5 ± 1.5	4.9 ± 2.2	<0.001
IgM (g/L)	1.6 (1.0,2.5)	1.5 (1.0,2.3)	0.717
WBC (10^9^/L)	5.6 ± 6.0	3.9 ± 2.3	0.019
RBC (10^12^/L)	4.9 ± 11.1	3.2 ± 0.7	0.183
HGB (g/L)	117.0 ± 19.7	100.9 ± 23.6	<0.001
INR	1.2 (1.0,1.4)	1.3 (1.1,1.5)	<0.001
PTA (%)	75.2 (62.3,94.8)	61.5 (50.0,76.0)	<0.001
PLT (10^9^/L)	163.5 ± 72.9	89.7 ± 56.9	<0.001
NLR	2.4 ± 3.2	2.7 ± 1.9	0.485
Other autoimmune diseases	27 (23.3%)	16 (20.0%)	0.216
Simplified score system	6.2 ± 0.5	6.1 ± 0.2	0.055
TG (mmol/L)	1.5 ± 0.7	0.9 ± 0.5	<0.001
TC (mmol/L)	3.8 ± 1.7	3.0 ± 1.0	0.001
HDL-C (mmol/L)	0.8 ± 0.6	0.7 ± 0.5	0.166
LDL-C (mmol/L)	3.5 ± 15.6	1.6 ± 0.7	0.030

*ANA* Anti-nuclear antibodies, *SMA* anti-smooth muscle antibodies, *ALT* Alanine aminotransferase, *AST* Aspartate aminotransferase, *TBIL* Total bilirubin, *GGT* Gamma-glutamyltranspeptidase, *IgG* Immunoglobulin G, *IgA* Immunoglobulin A, *IgM* Immunoglobulin M, *WBC* White blood cells, *RBC* Red blood cells, *HGB* Hemoglobin, *INR* International normalized ratio, *PTA* Prothrombin activity, *PLT* Platelets, *NLR* Neutrophil-lymphocyte ratio, *TG* Triglycerides, *TC* Total cholesterol, *HDL-C* High-density lipoprotein cholesterol, *LDL-C* Low density lipoprotein cholesterol

### Factors associated with AIH-related cirrhosis

Univariate and multivariate binary logistic regression analyses were performed to evaluate the factors associated with AIH-related cirrhosis. The results from the multivariate analysis presented in Table 2 show that patients with cirrhosis were older (age>50 years) than the noncirrhotic group. Patients with cirrhosis had elevated IgA (>4.35 g/L) and PLT (<100*10^9^/L) levels and lower ALT levels. We also included serum lipid metabolism indicators in the regression analysis and dichotomized them according to the optimal cutoff value. Univariate results showed lower serum TG (≤0.95 mmol/L), TC (<2.9 mmol/L), and LDL-C (≤1.48 mmol/L) levels in patients with cirrhosis. However, the results of the multifactorial regression analysis indicated that only TG levels were associated with AIH cirrhosis.

**Table 2:** Factors associated with cirrhosis in patients with AIH

	Univariate analysis		Multivariate analysis	
	OR (95%CI)	*P* values	OR (95%CI)	*P* values
Age>50 years	0.147 (0.065-0.332)	<0.001	7.682 (2.235-26.387)	0.001
Diabetes	0.591 (0.284-1.233)	0.161		
IgA>4.53 g/L	4.380 (2.327-8.242)	<0.001	3.170 (1.283-7.835)	0.012
WBC<4*10^9^/L	2.571 (1.431-4.618)	0.002		
RBC<3.5*10^12^/L	6.053 (3.223-11.366)	<0.001		
HGB<120 g/L	3.978 (2.173-7.282)	<0.001		
PLT<100*10^9^/L	7.875 (4.115-15.069)	<0.001	2.988 (1.127-7.919)	0.028
ALT>50 U/L	0.188 (0.099-0.357)	<0.001	0.176 (0.048-0.645)	0.009
AST>40 U/L	0.278 (0.134-0.574)	0.001		
ALB<40 g/L	5.353 (1.182-24.247)	0.030		
GGT>60 U/L	0.337 (0.181-0.629)	0.001		
TC<2.9 mmol/L	3.462 (1.857-6.455)	<0.001		
TG≤0.95 mmol/L	7.000 (3.704-13.230)	<0.001	3.036 (1.049-8.788)	0.041
LDLc≤1.48 mmol/L	0.333 (0.182-0.612)	<0.001		
INR>1.2	3.117 (1.710-5.679)	<0.001		
PTA<70%	2.829 (1.570-5.099)	0.001		

### Distribution of cirrhosis in subgroups

**Fig. 1:**
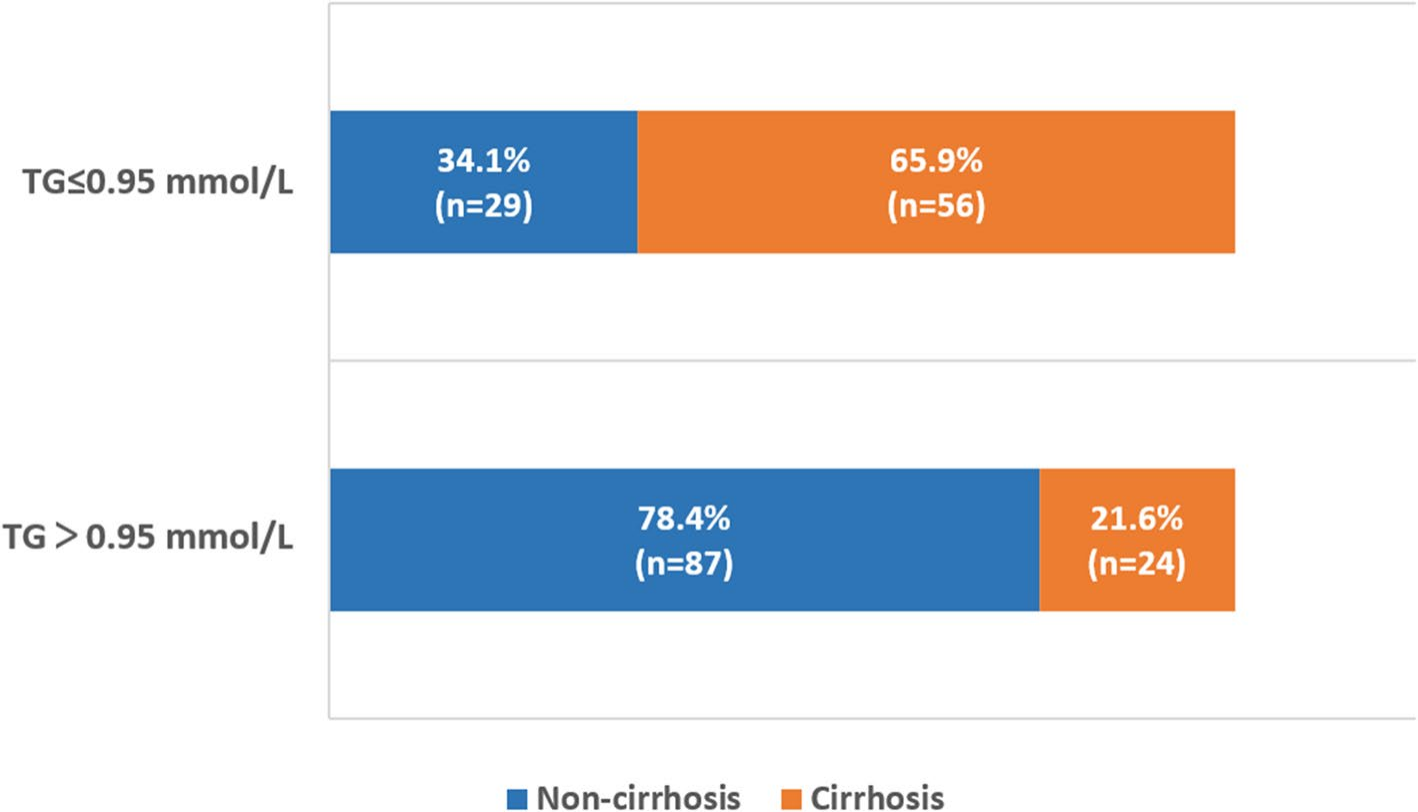
Distribution of liver cirrhosis and nonliver cirrhosis among patients with different triglyceride levels

Based on these results, we confirmed that the TG level is an important factor associated with cirrhosis, and then we continued to analyze the correlation between TG levels and cirrhosis among different subgroups. As shown in Fig. 1, more patients had cirrhosis (65.9%) in the TG≤0.95 mmol/L (*n*=85) group than in the TG>0.95 mmol/L group. We further divided patients into different subgroups according to age, PLT, IgA and ALT, and observed the distribution of patients with cirrhosis presenting different TG levels. As shown in Table 3, in the older age group (>50), more patients had cirrhosis in the TG≤0.95 mmol/L group than in the TG>0.95 mmol/L group (79% vs. 28%). Regardless of stratification by PLT, IgA and ALT levels, consistently more cirrhotic patients were included in the lower TG level group.

**Table 3:** Relationship between liver cirrhosis and TG levels in different subgroups

	Cirrhosis (*n*=80)	Cirrhosis in TG≤0.95 (*n*=56)	Cirrhosis in TG>0.95 (*n*=24)	*P* values
Age				
>50 years	72 (52%)	52 (79%)	20 (28%)	<0.001
≤50 years	8 (14%)	4 (21%)	4 (10%)	0.263
IgA				
>4.53 g/L	43 (64%)	30 (86%)	13 (41%)	<0.001
≤4.53 g/L	37 (29%)	26 (52%)	11 (14%)	<0.001
PLT				
>100*10^9^/L	54 (69%)	43 (81%)	11 (44%)	0.001
≤100*10^9^/L	26 (20%)	13 (41%)	13 (15%)	0.003
ALT				
>50 U/L	35 (27%)	22 (51%)	13 (15%)	<0.001
≤50 U/L	45 (67%)	34 (81%)	11 (44%)	0.002

### Correlation of serum TG levels with the severity of AIH-related cirrhosis

**Fig. 2:**
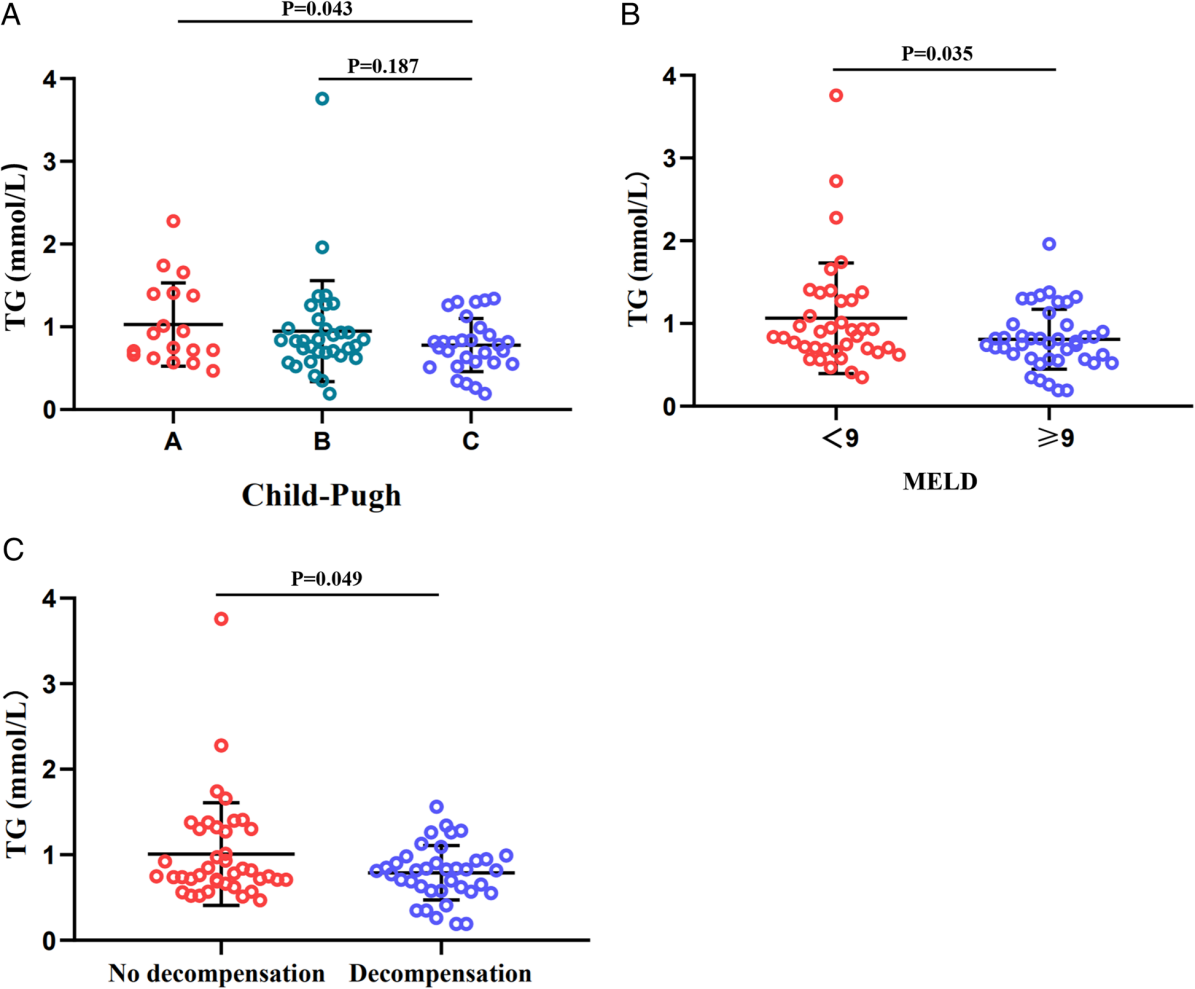
Triglyceride levels in patients with different degrees of cirrhosis based on the Child–Pugh classification (**A**), MELD score (**B**) and liver compensatory status (**C**)

Previous studies have suggested that lipid levels are related to the severity of cirrhosis, and thus we analyzed the relationship between serum TG levels and the severity of cirrhosis in patients with AIH. We compared the serum TG levels in patients with AIH cirrhosis at different stages of progression. Based on Child–Pugh scores, we divided the patients into three groups: Child A (*n*=18), Child B (*n*=33) and Child C (*n*=29). As shown in Fig. 2A, TG levels showed a decreasing trend in patients with Child–Pugh A, B and C, with mean values of 1.8 mmol/L, 1.3 mmol/L and 1.0 mmol/L, respectively. Compared to Group A, patients in Group C had significantly lower TG levels (*P*<0.05). Next, patients were divided into two groups according to the optimal cutoff MELD score. TG levels were compared across the different MELD classifications (Fig. 2B), and TG levels were lower in the higher MELD score (≥9) group than in the lower MELD score (<9) group (0.810 mmol/L vs. 1.063 mmol/L). In addition, compared to nondevised patients, the patients with decompensated cirrhosis had lower TG levels (0.891 mmol/L vs. 1.076 mmol/L) (Fig. 2C). We concluded that the worse the liver function, the lower the TG level.

### Effect of serum TG levels on the survival of patients with AIH-related cirrhosis

**Fig. 3:**
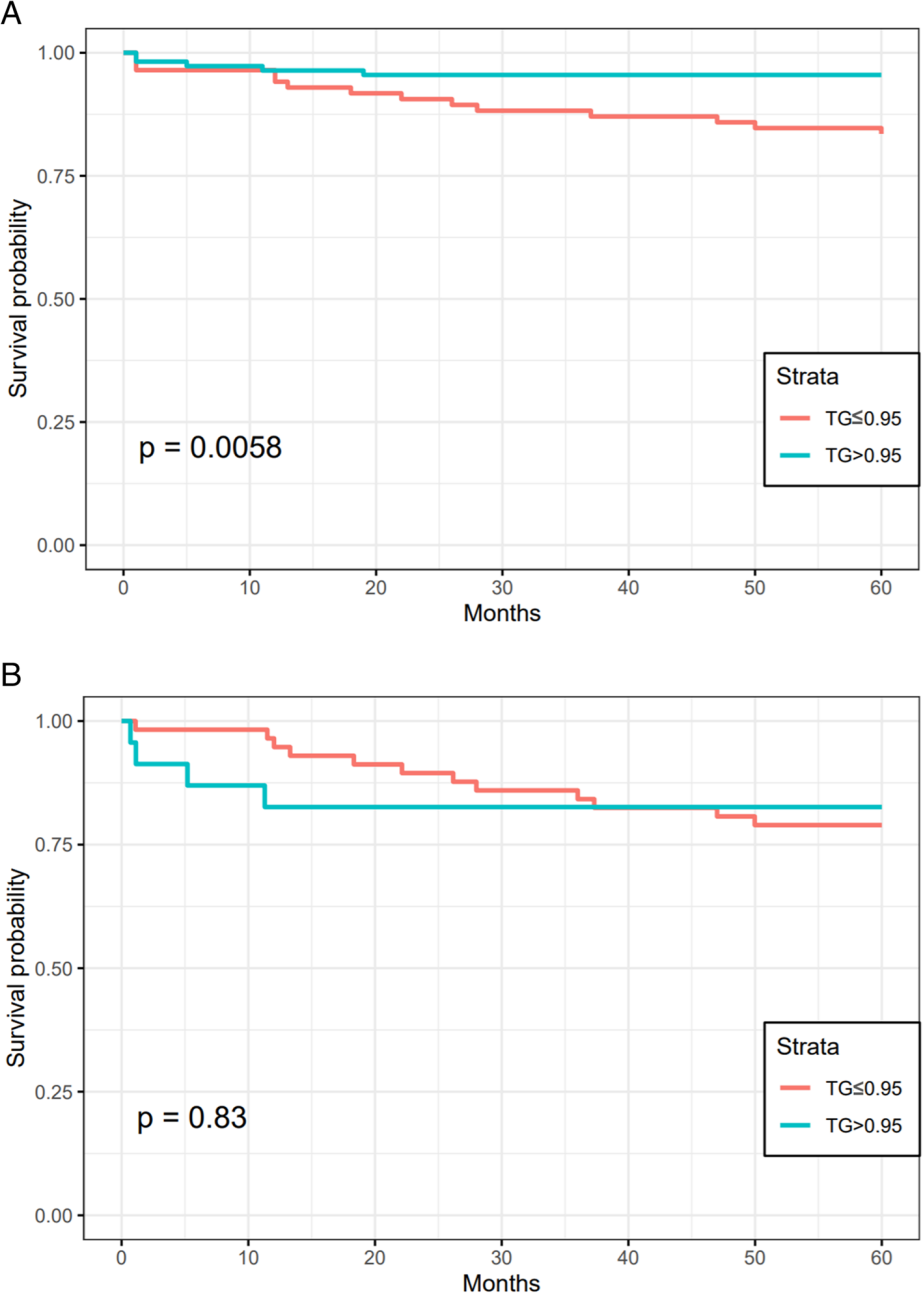
Overall survival of patients in both groups (TG level> 0.95 mmol/L and TG level≤ 0.95 mmol/L) from the total (**A**) and AIH cirrhosis (**B**) populations

**Fig. 4:**
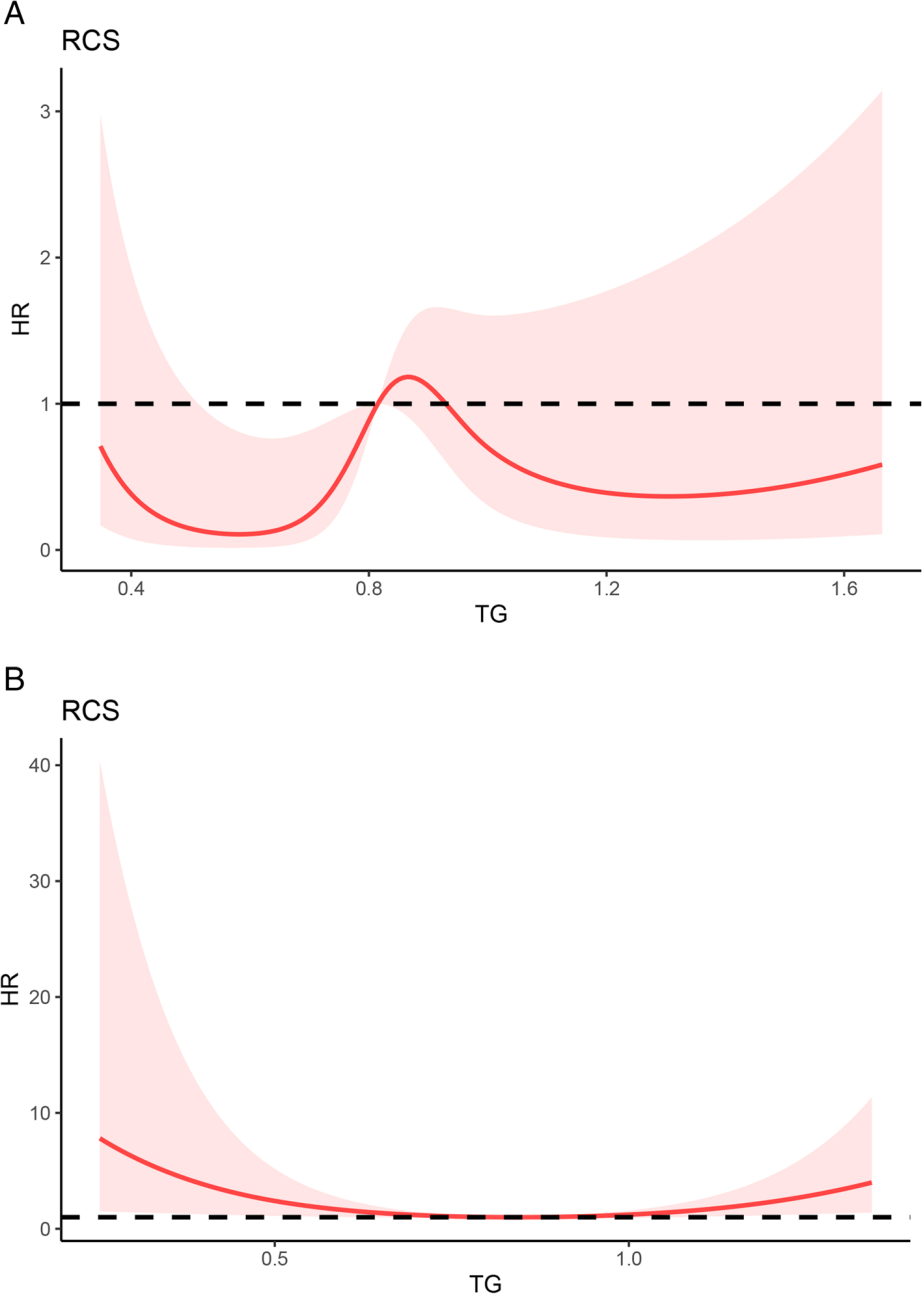
Association between triglyceride levels and overall survival of patients with AIH cirrhosis (**A**) and decompensated cirrhosis (**B**) using a restricted cubic spline regression model. HR indicates the hazard ratio

We further analyzed the role of TG levels in determining the prognosis of patients with AIH. During the 60-month follow-up (mean 56.05 months), 22 deaths occurred, including 18 patients with AIH cirrhosis. Figure 3A shows the 5-year survival rates of patients with different TG levels in the total AIH population, which were lower in the group of patients with TG levels ≤0.95 mmol/L (log-rank *P*<0.05) (HR=3.79, 95% CI: 1.528-9.423). However, in patients with AIH cirrhosis, no significant difference in 5-year OS was observed among groups with different TG levels (Fig. 3B). In Fig. 4, we used restricted cubic splines to flexibly construct the model and visualized the nonlinear relationship between predicted serum TG levels and overall survival in patients with cirrhosis. After fitting the Cox proportional risk model, five nodes were identified at the 5th, 35th, 50th, 65th and 95th percentiles of TG levels. The risk of death gradually increased in the interval of TG levels of 0.5-0.8 mmol/L(*P* for nonlinearity<0.001), and the hazard ratio per standard deviation increase in TG levels was 0.97 (95% CI: 0.94-0.99). The risk of death reached a maximum at 0.8 mmol/L, and then the curve was relatively flat at TG levels >0.8 (Fig. 4A). Furthermore, we observed a nonlinear relationship between TG levels and the overall survival of patients with decompensated cirrhosis. The results showed a U-shaped relationship between TG levels and survival (Fig. 4B). The restrictive cubic spline plot showed that the risk ratio progressively decreased within the lower TG levels, which reached a minimum risk of approximately 0.6 mmol/L and then gradually increased (*P* for nonlinearity=0.002). Below 0.6 mmol/L, the probability of TG risk per standard deviation prediction was 1.49 (95% CI: 1.00-2.24). The results suggested that lower TG levels were a risk factor for death in patients with AIH regardless of whether they had cirrhosis or decompensated cirrhosis.

## Discussion

Cirrhosis is an important risk factor affecting the outcome of patients with AIH. Most of the current reports on AIH cirrhosis investigate clinical management, diagnosis, treatment plan and prognosis, while lipid metabolism levels in patients with AIH cirrhosis have been less studied. Previous studies have confirmed that blood lipids including HDL-C, TG and TC, may be new indicators affecting the prognosis of patients with cirrhosis [8–10, 23], but most of these studies discussed patients with non-AIH cirrhosis, such as HBV, HCV and nonalcoholic fatty liver. Therefore, the present study observed the role of lipids in AIH cirrhosis. The results revealed that serum TG levels were potentially associated with the adverse outcomes of patients with AIH cirrhosis and may serve as a marker for end-stage liver disease.

In this study, we observed from the baseline data that patients with AIH cirrhosis had lower TC, TG and LDL-C levels. We later dichotomized TG levels according to the cutoff values. According to the results of cross-sectional multifactorial logistic regression analysis, a lower TG level was a factor associated with cirrhosis, suggesting that TG levels may be related to the development of cirrhosis in patients with AIH. Studies have shown that the liver can transform cholesterol or fatty acids absorbed from the diet and surrounding adipose tissue into lipoprotein complexes that are eventually released into the blood. As the biosynthetic capacity of lipoproteins decreases, the production of triglycerides and cholesterol in the blood will be reduced [24]. The uptake, synthesis and conversion of lipids are in dynamic balance in the absence of disease. When hepatocytes are damaged, such as during liver fibrosis and viral hepatitis, the reserves of the liver are reduced while lipid catabolism increases, ultimately causing nutritional deficiencies [25–27]. Patients with cirrhosis often have hypotriglyceridaemia, which may be caused by the decreased ability of the liver to synthesize lipoprotein [28]. In addition, age, IgA levels, PLT counts and liver function were also associated with cirrhosis in the AIH population included in this study, similar to the results of previous studies [29, 30].

Blood lipid levels were associated with the severity of cirrhosis in previous studies. Furthermore, the study analyzed the TG level in patients with AIH presenting with cirrhosis and found that TG levels were lower in patients with a higher Child–Pugh grade and MELD score, indicating that TG levels may be related to the progression of cirrhosis. A potential explanation for this finding is that liver function is also poor in patients with severe cirrhosis. Most of the triglycerides are derived from the diet, and a small percentage is self-synthesized. The liver converts the fatty acids in food into triglycerides and stores most of them in adipose tissue. The catabolism of triglycerides in fat cells provides energy to the body. Some studies have shown that malnutrition and fat loss occur in patients with advanced liver disease, thus reducing triglyceride and cholesterol levels in peripheral blood [24]. As the degree of cirrhosis increases, blood lipid levels decrease gradually [31], which might be considered a new indicator of liver disease prognosis. Next, we observed the effect of TG levels on the prognosis of patients with AIH cirrhosis. The KM curve showed that a lower level of TG was associated with shorter survival of all patients with AIH, and the restricted cubic spline described the results of a nonlinear relationship between TG levels and the overall survival of patients with cirrhosis. Patients with AIH cirrhosis who presented low triglyceride levels (0.5-0.8 mmol/L) had a greater risk of death. TG, a lipid metabolism indicator, has been considered strongly associated with adverse outcomes of patients with liver cirrhosis or HCC in several studies. A retrospective study showed an association between TG levels and different Child–Pugh classifications of alcoholic cirrhosis [27]. Another cross-sectional study showed that lower TG levels affect the degree of damage in patients with liver cancer presenting with cirrhosis [32]. Liu et al. suggested that low TG levels affect the overall survival and relapse-free survival of patients with HCC [12]. Furthermore, the observation that lower TG levels in patients with decompensated cirrhosis remain a prognostic risk factor is interesting.

### Comparisons with other studies and what the current work adds to the existing knowledge

Previous studies have addressed the relationship between lipid metabolism and non-AIH cirrhosis, including viral hepatitis, and nonalcoholic fatty liver [8–10, 23]. To our knowledge, this study is the first to evaluate TG levels in a Chinese population of patients with AIH-related cirrhosis. The few studies examining the relationship between lipid levels and AIH are limited to prevalence surveys and have relatively small sample sizes [13]. The present study not only analyzed the relationship between lipid levels and AIH cirrhosis but also found that TG levels were associated with end-stage liver disease. This work aptly complements the current study. Moreover, the effect of lipids on the long-term outcomes of patients with AIH is unknown. Relevant studies have shown that lipid indicators are important factors affecting short- and long-term mortality in patients with HBV-related decompensated cirrhosis [7, 10, 33]. Our study focuses on the population with AIH-related cirrhosis and provides new evidence for the effects of lipids on the outcome of cirrhosis, which provides a basis for the identification of patients who might have a poor prognosis and has an important reference value for the adjustment of treatment strategies.

### Study strengths and limitations

Strengths of this study: This study analyzed the relationship between lipid levels and AIH cirrhosis, complementing the value of metabolism levels in patients with cirrhosis of different etiologies. In addition, the study revealed that TG levels are associated with advanced liver disease in patients with AIH and predict the long-term prognosis of patients. However, this study has some limitations. First, this study employs a retrospective design and has missing data such as baseline height or weight information of patients, which may cause bias. Second, due to the uncommon nature of AIH and the inclusion of only patients from a single center, the sample size of patients with AIH cirrhosis in this study was small and the results may need to be further validated by expanding the sample size in the future. Third, the lack of patient treatment strategy among the included variables may be an important factor affecting the outcome, and the effect of treatment modality on lipid levels must be further clarified in a follow-up study. Fourth, the population in this study was limited to the Asian population, and further confirmation is needed among other races.

## Conclusions

In conclusion, this study confirmed the effects of blood lipid levels on patients with AIH. Low TG levels were related to advanced liver disease while predicting the long-term survival of patients. TG is a clinically easily detectable biochemical index that may be used as a new indicator of end-stage liver disease or the long-term prognosis of patients with AIH. In summary, we concluded that close monitoring of blood lipid levels in patients with AIH cirrhosis and timely improvement of treatment strategies may better prevent cirrhosis and reduce the occurrence of fatal events, which provides a new treatment method for patients with AIH and has an important reference value. More clinical and mechanistic studies are needed in the future to further validate the relationship between metabolism and AIH cirrhosis.

## Data Availability

Our study was a retrospective cohort study, which only collected the clinical data of patients, did not interfere with the treatment plan of patients, and did not bring risks to the physiology of patients. The researchers will try their best to protect the information provided by patients from disclosing personal privacy.
